# Inhibition of Caspase-1 Ameliorates Ischemia-Associated Blood-Brain Barrier Dysfunction and Integrity by Suppressing Pyroptosis Activation

**DOI:** 10.3389/fncel.2020.540669

**Published:** 2021-01-11

**Authors:** Yubin Liang, Pingping Song, Wei Chen, Xuemin Xie, Rixin Luo, Jiehua Su, Yunhui Zhu, Jiamin Xu, Rongrong Liu, Peizhi Zhu, Yusheng Zhang, Min Huang

**Affiliations:** ^1^Department of Neurology, Zhuhai People’s Hospital, Zhuhai Hospital Affiliated with Jinan University, Zhuhai, China; ^2^Department of Neurology and Stroke Center, The First Affiliated Hospital of Jinan University, Guangzhou, China; ^3^Clinical Neuroscience Institute, Jinan University, Guangzhou, China; ^4^Department of Stroke Center, GuangZhou Panyu Central Hospital, Guangzhou, China; ^5^Department of Neurology, The Seventh Affiliated Hospital, Sun Yat-sen University, Shenzhen, China

**Keywords:** ischemic stroke, caspase-1, blood-brain barrier, pyroptosis, RAGE, MAPK

## Abstract

Ischemic cerebral infarction represents a significant cause of disability and death worldwide. Caspase-1 is activated by the NLRP3/ASC pathway and inflammasomes, thus triggering pyroptosis, a programmed cell death. In particular, this death is mediated by gasdermin D (GSDMD), which induces secretion of interleukin (IL)-1β and IL-18. Accordingly, inhibition of caspase-1 prevents the development and worsening of multiple neurodegenerative diseases. However, it is not clear whether inhibition of caspase-1 can preserve blood-brain barrier (BBB) integrity following cerebral infarction. This study therefore aimed at understanding the effect of caspase-1 on BBB dysfunction and its underlying mechanisms in permanent middle cerebral artery occlusion (MCAO). Our findings in rat models revealed that expression of caspase-1 was upregulated following MCAO-induced injury in rats. Consequently, pharmacologic inhibition of caspase-1 using vx-765 ameliorated ischemia-induced infarction, neurological deficits, and neuronal injury. Furthermore, inhibition of caspase-1 enhanced the encapsulation rate of pericytes at the ischemic edge, decreased leakage of both Evans Blue (EB) and matrix metalloproteinase (MMP) proteins, and upregulated the levels of tight junctions (TJs) and tissue inhibitors of metalloproteinases (TIMPs) in MCAO-injured rats. This in turn improved the permeability of the BBB. Meanwhile, vx-765 blocked the activation of ischemia-induced pyroptosis and reduced the expression level of inflammatory factors such as caspase-1, NLRP3, ASC, GSDMD, IL-1β, and IL-18. Similarly, vx-765 treatment significantly reduced the expression levels of inflammation-related receptor for advanced glycation end products (RAGE), high-mobility family box 1 (HMGB1), mitogen-activated protein kinase (MAPK), and nuclear factor-κB (NF-κB). Evidently, inhibition of caspase-1 significantly improves ischemia-associated BBB permeability and integrity by suppressing pyroptosis activation and the RAGE/MAPK pathway.

## Introduction

Ischemic stroke is a major cause of death and disability in adults worldwide and severely affects human health and constrains subsequent rehabilitation (Cohen and Leker, 2011). Studies showed that blood-brain barrier (BBB) destruction is one of the most important consequences of cerebral infarction (Moretti et al., 2015). Pathophysiologically, ischemic stroke destroys BBB to varying degrees, ranging from primary to secondary to ultimate death of microvascular endothelial cells (ECs; Krueger et al., 2015). Moreover, BBB is a highly specific endothelial tissue whose function is regulated by brain microvascular ECs. It prevents recruitment of peripheral blood leukocytes into the brain parenchyma (Krueger et al., 2015). Various factors impair BBB function after cerebral infarction. Meanwhile, microvascular endothelium, adherens junction (AJs), pericyte, and tight junction (TJs) proteins such as zonula occludens-1 (ZO-1) and occludins play important roles in blocking recruitment of peripheral leukocytes. They also regulate the BBB function after cerebral infarction (Yang et al., 2013). Cognizant of this, BBB is undoubtedly a potential therapeutic target in cerebral infarction.

Numerous studies have implicated inflammation in cerebral infarction (Tuttolomondo et al., 2014; Kawabori and Yenari, 2015; Tong et al., 2015). In particular, it destroys the BBB, resulting in secondary damage that increases the risk of hemorrhagic transformation. It further hinders the recovery of peri-infarct regions. As such, inhibiting inflammation can potentially enhance BBB recovery after cerebral infarction. A large number of endogenous risk signals such as adenosine triphosphate (ATP), reactive oxygen species (ROS), and cathepsin are produced following brain ischemia injury (Jorgensen and Miao, 2015). The corresponding endogenous damage signals are recognized as a damage-associated molecular pattern (DAMP), which initiates a series of signal cascades that activate pro-caspase-1. This aggravates the release of lots of pro-inflammatory cytokines such as interleukin (IL)-1β and IL-18, which together trigger pyroptotic cell death (Malik and Kanneganti, 2017; Nagata and Tanaka, 2017). Expression of caspase-1 significantly increases in neurons after ischemic stroke. Consequently, inhibition of caspase-1 is neuroprotective against middle cerebral artery occlusion (MCAO) damage (Rabuffetti et al., 2000; Li et al., 2019). For instance, a mouse model showed that caspase-1 knockout significantly attenuated cerebral ischemic injury. Additionally, inhibition of caspase-1 induces anti-inflammatory and anti-apoptotic processes, shown to alleviate neuronal damage in the hippocampal CA1 region after cerebral infarction (Hara et al., 1997; Ross et al., 2007; Zhao et al., 2017). This strongly underlines the role of caspase-1 in cell death after ischemic stroke. However, the role of caspase-1-mediated BBB dysfunction in cerebral infarction remains unknown.

The receptor for advanced glycation end products (RAGE) is a member of the immunoglobulin superfamily. The superfamily increases the severity of the inflammatory response by binding RAGE ligands (Yatime and Andersen, 2014), such as advanced glycation end products (AGE), S100 proteins, the high-mobility family box-1 (HMGB1) protein, and β-amyloid fibrils (Sirois et al., 2013). Thus, RAGE activates HMGB1 signaling pathways to prompt caspase-1 activation and downstream production of various inflammation mediators (Yan et al., 2012). Moreover, inhibition of RAGE alleviates BBB leakage and brain edema. It also increases the expression of TJ scaffold proteins but downregulates matrix metalloproteinase (MMP)-2 and MMP-9 proteins (Wan et al., 2015). Furthermore, RAGE plays a significant role in immune response and regulation of inflammation through the nuclear factor-κB (NF-κB). NF-κB is a downstream signaling pathway molecule of RAGE (Vicente Miranda and Outeiro, 2010), activated following binding HMGB1 to its corresponding receptor, RAGE (Lv et al., 2009). Thus, rat models show that inhibiting RAGE alleviates cerebral infarction damage in rats *via* the NF-κB-mediated inflammatory pathway (Ning et al., 2014). Mitogen-activated protein kinase (MAPK) is also a crucial signaling pathway in the pathological process of ischemic stroke. It modulates the effects of cerebral infraction by destroying the BBB and activation of inflammatory mediators (Nito et al., 2008; Mohamed et al., 2015).

This study therefore aimed at validating the effects of inhibiting caspase-1 on BBB integrity in rats after MCAO. The study further explored mechanisms underlying caspase-1-induced BBB damage, as well as the precise pathology of ischemic stroke.

## Materials and Methods

### Permanent MCAO Model

All experiments were conducted in line with the guidelines of the Animal Care Committee of Jinan University. The protocols for this research were approved by the Animal Ethics Committee of Jinan University. We used 3-month-old Sprague–Dawley rats weighing between 300 and 350 g and sourced from Guangdong Medical Laboratory Animal Center (certificate number: SYXK2017-0174). Ischemia was induced by MCAO. Briefly, the rats were first anesthetized by intraperitoneal injection of 10% chloral hydrate (3 ml/kg; cat# 302-17-0, BBI Life Sciences, Shanghai, China). The left distal branch of the middle cerebral artery was then exposed by subtemporal craniectomy, guided by an operating microscope. MCAO was performed using bipolar electrocoagulation, resulting in a permanent focal cerebral infarction. Sham animals underwent the same operating procedures but did not undergo electrocoagulation. In total, 150 rats underwent MCAO while the remaining 44 served as sham-operated controls. During surgery and throughout the recovery period, the body temperatures of all rats were maintained at 37 ± 0.5°C, and they were provided with adequate water and food.

### vx-765 Treatment

Here, 1 h after permanent MCAO, the rats were intraperitoneally injected either with 10% DMSO in normal saline (as a control), once a day for 3 days, or with vx-765 (cat# 273404-37-8, 25 mg/kg, MedChemExpress; 50 or 100 mg/kg), sourced from Vertex Pharmaceuticals as previously described (Ravizza et al., 2006). These procedures were not performed on sham-operated rats.

### Neurobehavioral Evaluation

Neurological deficiency was assessed using the Garcia test, forelimb placing test, and rotarod behavioral test (Garcia et al., 1995; Hua et al., 2002) at 6 h, 12 h, 1 day, 2 days, and 3 days after cerebral infarction. Neurological evaluation encompassed six parameters: body symmetry, autonomic activity, forelimb extension, screen experiments, beard tactile reflections, and bilateral sensory reflexes. Tests were performed in a blinded fashion, with assessments done without prior knowledge of which group the rats belonged to.

### 2,3,5-Triphenyltetrazolium Chloride (TTC) Staining

The rats were deeply anesthetized using 10% chloral hydrate (i.p.) and then decapitated after MCAO. The brains were removed, sliced into mold, and sliced into 3-mm-thick coronal sections, and kept for 30 min at 37°C in 1% 2,3,5-Triphenyltetrazolium Chloride (TTC) solution (cat# 298-96-4, BBI Life Sciences, China). Thereafter, the tissues were fixed with 4% paraformaldehyde (PFA, cat# G1101, Servicebio, Guangzhou, China) for 24 h at 4°C. The brain sections were photographed using a digital camera, and the infarct volume was determined using the ImageJ software (National Institutes of Health).

### Western Blotting

The amount of peri-infarcted protein levels was detected and analyzed using western blotting, based on the BCA protein assay kit (cat# ab102536, Abcam, USA). Briefly, 20 μg of brain proteins was then separated using SDS-PAGE and electro-transferred onto PVDF membranes following manufacturer’s instructions (cat# IPVH00010, Millipore, USA). The membranes were then blocked for 2 h at room temperature (RT) with 5% nonfat milk (cat# A600669, BBI, Shanghai, China). Thereafter, the membranes were then incubated for 24 h at 4°C with multiple antibodies: cleaved caspase-1 (1:1,000, cat# 89332, Cell Signaling Technology), NeuN (1:1,000, cat# 23407, CST), NLRP3 (1:1,000, cat# DF7438, Affinity), ASC (1:1,000, cat# ab47092, Abcam), gasdermin D (GSDMD, 1:500, cat# sc-393581, Santa Cruz), MMP-1 (1:500, cat# ab137322, Abcam), MMP-2 (1:500, cat# wl03702, Wanleibio), MMP-3 (1:500, cat# ab52915, Abcam), MMP-9 (1:3,000, cat# ab228405, Abcam), tissue inhibitor of metalloproteinases (TIMP)-1 (1:500, cat# wl02342, Wanleibio), TIMP-2 (1:500, cat# wl02343, Wanleibio), ZO-1 (1:2,000, cat# ab221547, Abcam), occludin (1:2,000, cat# ab216327, Abcam), claudin-3 (1:1,000, cat# ab133187, Abcam), claudin-5 (1:2,000, cat# ab131259, Abcam), glucose transporter protein-1 (GLUT-1, 1:2,000, cat# 07-1401, Sigma), osteopontin (OPN, 1:1,000, cat# sc-21721, Santa Cruz), RAGE (1:1,000, cat# 42544, CST), HMGB1 (1:3,000, cat# ab18256, Abcam), AGE (1:2,000, cat# ab176173, Abcam), S100B (1:500, cat# ab52642, Abcam), P38 (1:1,000, cat# 8690, CST), p-P38 (1:1,000, cat# 4511, CST), ERK (1:1,000, cat# 4695, CST), p-ERK (1:1,000, cat# 4370, CST), NF-κB (1:1,000, cat# ab32360, Abcam), p-NF-κB (1:1,000, cat# 59674, CST), IκBa (1:1,000, cat# 4812, CST), p-IκBa (1:1,000, cat# 9241, CST), JNK (1:1,000, cat# 3708, CST), p-JNK (1:1,000, cat# 4668, CST), and β-actin (1:3,000, cat# 4970, CST). The membranes were further incubated with horseradish peroxidase (HRP)-linked goat anti-rabbit IgG (1:2, 000, cat# ab6721, Abcam) or goat anti-mouse IgG secondary antibodies (1:2,000, cat# ab6789, Abcam). The presence and amount of proteins were detected and assessed using the enhanced Tanon 2500 system (Tanon, China). The blot images were then processed and analyzed using the ImageJ software (NIH, USA).

### Tissue Preparation and Immunofluorescence Staining

Briefly, each rat was intraperitoneally injected with 10% chloral hydrate and then perfused with 150 ml of 0.9% saline and later with 250 ml of 4% PFA in 0.1 M phosphate buffer (pH = 7.4) at 4°C. Brain tissues were isolated from the rats and preserved for 3 days at 4°C in 4% PFA. The tissues were then embedded in paraffin and cut into 3-mm-thick sections for immunofluorescence microscopy. After being mounted onto glass slides, and brain tissue sections were treated using 0.01 mol/L of citrate buffer at 85°C (pH = 6.0). The slides were then washed thrice with phosphate-buffered saline (PBS) and blocked for 1 h with 5% goat serum. Thereafter, they were incubated for 24 h at 4°C with multiple primary antibodies: cleaved caspase-1 p20 (1:50, cat# sc-398715, Santa Cruz), NeuN (1:500, cat# 24507, CST), desmin (1:100, cat# 32362, Abcam), CD31 (1:200, cat# ab24590, Abcam), and RAGE (1:00, cat# ab216329, Abcam), followed by incubation with Alexa Fluor 488 (1:100, cat# ab150077, Abcam) and Alexa Fluor 695 (1:100, cat# ab150116, Abcam) secondary antibodies, with 4′,6-diamidino-2-phenylindole (DAPI; cat# ab104139, Abcam) staining. The tissues were then analyzed using a fluorescence microscope (Leica, Heidelberger, Germany).

### Evans Blue (EB) Leakage Test and Brain Edema Volume

Permeability of BBB was assessed using the EB leakage test. Here, 4 ml/kg of 2% EB solution (cat# E2129, Sigma) was injected into the rat’s tail vein after 24 h of deep anesthesia. Both the left and right hemisphere tissues of the brain were then collected and soaked in formamide solution (1 ml/100 mg) for 24 h and centrifuged at 12,000 rpm for 10 min at 4°C. Two hundred microliters of the tissue supernatant was pipetted into a 96-well plate, and their optical density (OD) values were determined using a microplate reader at a wavelength of 632 nm (Chang et al., 2017). This experiment was replicated thrice, with the average of the readings used in subsequent analyses. The percentage of brain edema was then calculated as previously described (Gao et al., 2020).

### EZ-Link Sulfo-NHS-Biotin Leakage Testing

First, EZ-Link Sulfo-NHS-Biotin was perfused through the hearts of the live rats for 5 min, followed by a second perfusion with 200 ml of PFA. Brain tissues were then excised and immersed in PFA. They were then fixed for 1 day at 4°C. The brain tissues were then sliced into 10-mm-thick coronal sections and placed sequentially in 10%, 20%, and 30% sucrose for gradient dehydration at 4°C. The immersion was sustained until a level of gradient dehydration equilibrium was reached. The tissues were then embedded in O.C.T. (Sakura, #4583, USA). The embedded tissues were sliced into 10-μm-thick sections and washed for 30 min with 0.1 mol/L of PBS. They were then blocked for 1 h at RT using 5% goat serum and embedded onto sterile glass slides. Thereafter, the tissue sections were then incubated for 24 h at 4°C with primary antibodies anti-vWF (1:200, cat# ab194405, Abcam) and FITC–streptavidin (1:100, cat# A0556, Beyotime Biotechnology). After three washes with PBS, they were stained for 1 h in the dark with Cy3 rabbit anti-sheep IgG secondary antibody (1:100, cat# ab6939, Abcam). Finally, counterstaining was performed with DAPI, after which the tissues were observed under an immunofluorescent confocal microscope (Leica, Heidelberger, Germany).

### Enzyme-Linked Immunosorbent Assay (ELISA)

The levels of expression of IL-1β and IL-18 in the supernatant of peri-infarcted brain tissues were determined using rat IL-1β and IL-18 ELISA kits (cat# ab235646 and cat# ab213909, Abcam). The standard concentration range of the samples was linear between 5.47 and 350 pg/ml and between 15.6 and 1,000 pg/ml. Each experimental sample was tested thrice, with the average value used in subsequent analyses.

### Real-Time Polymerase Chain Reaction (RT-PCR)

Total RNA was extracted from peri-infarcted brain tissues of the rats using the TRIzol reagent (cat# 9108, Takara) following the manufacturer’s instructions. cDNA was then synthesized based on the HiFiScript cDNA Synthesis Kit (cat# CW2569, CWBIO). The caspase-1 and β-actin genes were then detected using the Bio-Rad iQ™ 5 multicolor RT-PCR detection system (USA). All reactions were performed in triplicate and normalized to β-actin following the 2^−ΔΔCt^ way. The primer pairs used were as follows: 5′ACAAGGCACGGGACCTATG 3′ and 5′ TCCCAGTCAGTCCTGGAAATG 3′ for forward and reverse primers, respectively, for caspase-1 gene, and 5′ GAGTACAACCTTCTTGCAGCTC 3′ and 5′ CATACCCACCATCACACCCTG 3′ for forward and reverse primers, respectively, for β-actin gene.

### Electron Microscopy

The rats were intraperitoneally administered with 10% chloral hydrate and then perfused with 0.1 M of cold PBS and thereafter with 2.5% glutaraldehyde. The brain tissues were embedded in epoxy resin and then observed using a HITACHI transmission electron microscope (HITACHI, Tokyo, Japan) at 80 kV. The samples were extracted at the peri-infarct regions of the brain.

### Statistical Analysis

Continuous data were expressed as means ± standard error of the mean (SEM), whereas the statistical differences among groups were evaluated using the one-way ANOVA, using the GraphPad Prism software (GraphPad Prism Software Inc., San Diego, CA, USA). A *P*-value of less than 0.05 was considered statistically significant.

## Results

### Time Course of Caspase-1 Expression After Permanent MCAO in Cerebral Infarction

**Figure 1:**
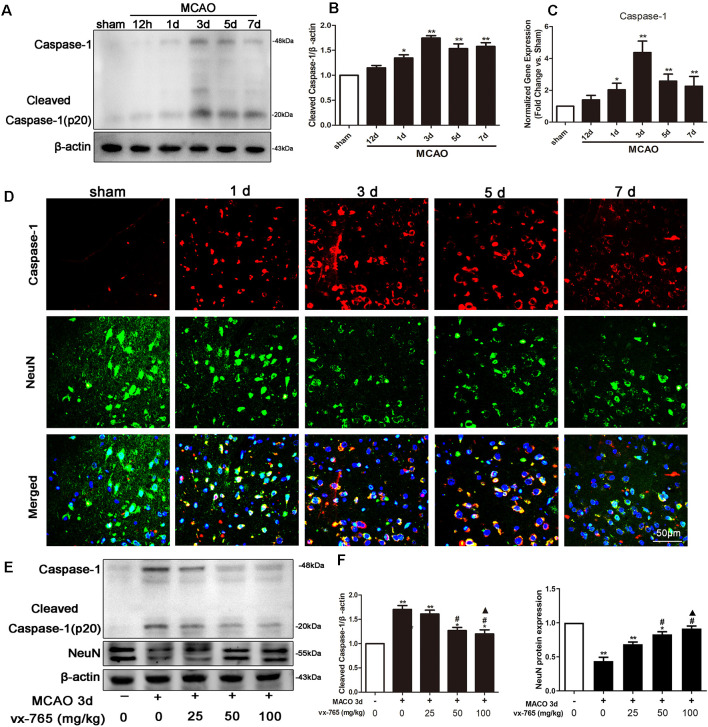
Middle cerebral artery occlusion (MCAO)-induced caspase-1 expression in rats. **(A,B)** Western blotting analysis of the expression of caspase-1 protein in the peri-infarct area. **(C)** RT-qPCR of caspase-1 mRNA at 12 h to day 7 after MCAO. **(D)** Representative images of fluorescent staining of caspase-1 (red) and the neuron marker (NeuN, green) in the ipsilateral thalamus after MCAO from day 1 to day 7. Scale bar = 50 μm. **(E)** Western blot analysis of caspase-1 and NeuN proteins at different concentrations of vx-765. **(F)** Combined western blotting statistics. All data are expressed as means ± SEM, *P < 0.05 and **P < 0.01, vs. sham, ^#^*P* < 0.05, vs. vehicle; ^▲^*P* > 0.05, vs. vx-765 50 mg/kg, *n* = 4 per group.

The levels of expression of caspase-1 at different time points after permanent MCAO are shown in Figure 1A. Here, western blotting results revealed that the expression of caspase-1 slightly increased in the experimental group at 12 h after MCAO, peaking at day 3. However, compared to the expression levels in the sham-operated group, they gradually decreased (Figures 1A,B; *P* < 0.05). qRT-PCR revealed comparable findings (Figure 1C; *P* < 0.05). Meanwhile, immunoreactivity of caspase-1 at 3 days after focal cortical infarction appeared mostly in cells with elongated, irregular nuclei and which were NeuN positive, as revealed by double immunostaining (Figure 1D). This implies that caspase-1 in the peri-infarct regions was predominantly localized in neurons at 3 days after MCAO. Notably, compared with 25 mg/kg of vx-765, 50 and 100 mg/kg doses significantly suppressed caspase-1 (*P* < 0.05). However, there was no significant difference between the 50 and 100 mg/kg vx-765 doses (Figures 1E,F; *P* > 0.05). Similarly, vx-765, an inhibitor of caspase-1, significantly rescued the decrease in the expression of neuronal protein following MCAO (Figures 1E,F; *P* < 0.05). Based on drug safety, economic benefits, and efficacy, a vx-765 drug concentration of 50 mg/kg was used for subsequent experiments.

### vx-765 Ameliorated Neurological Function Deficits and Decreased the Degree of Cerebral Infarction After MCAO

**Figure 2:**
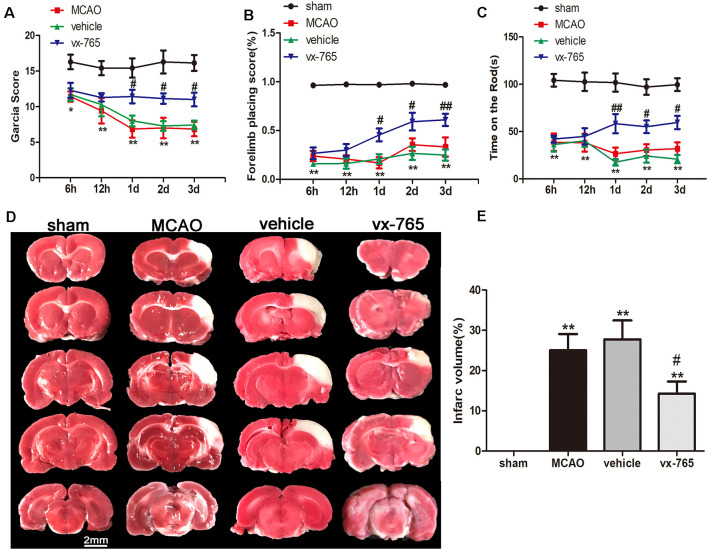
Caspase-1 inhibitor vx-765 reduced cerebral infarct volume and improved neurological deficits in rats following MCAO. **(A–C)** Neurological deficit scores estimated using Garcia scores, forelimb placing scores, and time of rod from 6 h to day 3 after MCAO. *n* = 35 per group. **(D)** 2,3,5-Triphenyltetrazolium Chloride (TTC) staining of the infarct area at day 3 after MCAO. **(E)** Corresponding statistical analyses of the infarct area. Scale bar = 2 mm, *n* = 3 per group. Data are shown as means ± SEM, *P < 0.05 and **P < 0.01, vs. sham; ^#^*P* < 0.05 and ^##^*P* < 0.01, vs. vehicle.

The Garcia test, forelimb placing test, and rotarod behavioral test were performed after cerebral infarction to assess sensory, motor ability, and limb reflex and balance, revealing that at 1 day after cerebral infarction, there were significant neurological declines in the vehicle animals and vx-765-treated animals compared to controls (Figure 2; *P* < 0.05). The neurological grading scores of the vehicle animals and vx-765-treated animals were significantly low compared to those of controls (sham-operated group). However, based on neurological scores, vx-765 administration significantly increased neural function at day 3 after ischemic stroke (Figures 2A–C; *P* < 0.05). Strikingly, TTC staining results revealed that compared to the vehicle animals, vx-765-treated animals had a significantly reduced infarct volume after MCAO (Figures 2D,E; *P* < 0.05).

### vx-765 Improved the Permeability of BBB and Brain Edema Following Cerebral Infarction

**Figure 3:**
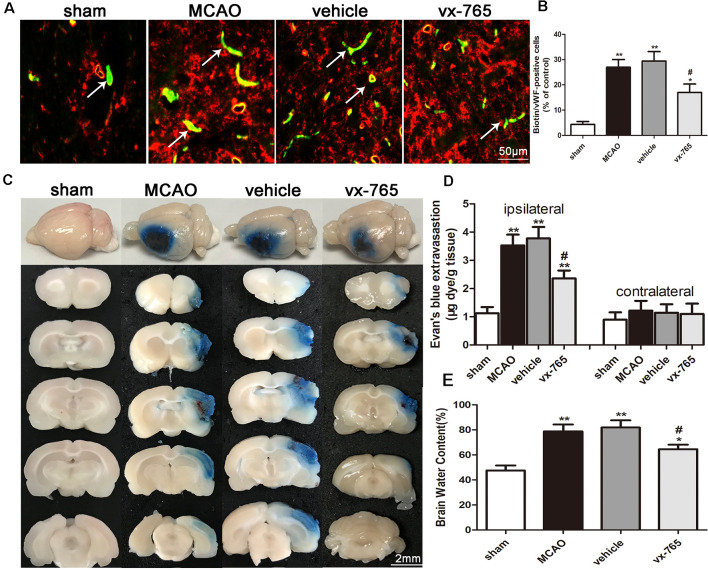
Blood-brain barrier (BBB) permeability and brain edema in rats at day 3 after vx-765 inhibition of caspase-1, following brain ischemia. **(A)** Immunofluorescence staining after Sulfo-NHS-Biotin tracer extravasation assay. The leakage of biotins (green) from vessels and vWF-positive cells (red) is indicated in the peri-infarct zone. **(B)** The percentage of biotin/vWF-positive cells. Scale bar = 50 μm. **(C)** The permeability of BBB into focal cortex tissues at the peri-infarct area estimated using Evans Blue (EB) assay. Scale bar = 2 mm. **(D)** Quantitative analysis of EB leakage rate in ipsilateral and contralateral regions. **(E)** The volume of brain edema on day 3 after MCAO. Data are expressed as means ± SEM, *P < 0.05 and **P < 0.01, vs. sham; ^#^*P* < 0.05, vs. vehicle, *n* = 5 per group.

Increased leakage of the exogenous tracer Sulfo-NHS-Biotin, as an indicator of BBB integrity, was observed in vWF-positive cells of MCAO rats after ischemia. In contrast, Sulfo-NHS-Biotin leakage decreased in vWF-positive cells of the vx-765 groups, indicative of less BBB disruption (Figures 3A,B; *P* < 0.05). Furthermore, EB staining revealed that BBB permeability increased significantly after MCAO. However, compared to the vehicle group, the vx-765-treated group showed reduced EB extravasation (Figure 3C; *P* < 0.05). Notably, there was no obvious change on EB leakage among the four groups with a normal hemisphere (Figures 3C,D). Similarly, brain edema was severe in the model and vehicle groups compared to that in the control group. However, vx-765 treatment significantly reduced the degree of brain edema compared to the vehicle treatment (Figure 3E; *P* < 0.05). These findings suggested that vx-765 ameliorated the permeability of BBB and reduced brain edema after MCAO.

### vx-765 Increased the Expression of TJ Proteins in Peri-infarct Regions

**Figure 4:**
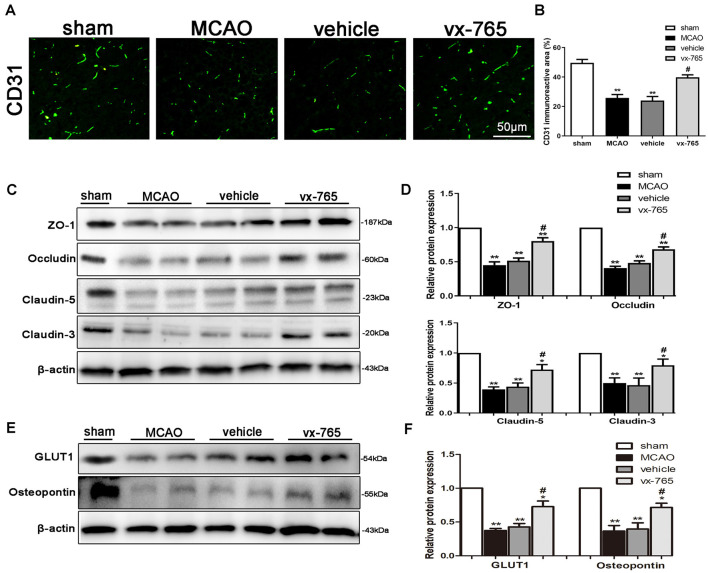
Expression of tight junction (TJ) proteins after caspase-1 inhibition by vx-765, following ischemic stroke. **(A)** Immunofluorescence staining of CD31 (green) in the peri-infarct regions. Scale bar = 50 μm. **(B)** Fluorescence intensity and corresponding statistics. **(C)** Western blotting of the amount of zonula occludens-1 (ZO-1), occludin, and claudin-5 proteins in the peri-infarct area and **(D)** corresponding statistics. **(E)** Immunoblot images of the expression of glucose transporter protein-1 (GLUT-1) and osteopontin (OPN) in the peri-infarct regions, with** (F)** corresponding statistical analyses. Data are expressed as means ± SEM, *P < 0.05 and **P < 0.01, vs. sham; ^#^*P* < 0.05, vs. vehicle, *n* = 6 per group.

Endothelial-specific markers (CD31) can maintain the integrity of BBB after MCAO (Cao et al., 2016; Wang et al., 2019). In this study, CD31 (green) significantly impacted BBB integrity after ischemic stroke. Immunofluorescent assessment revealed that CD31 was downregulated in the infarct area. However, vx-765 treatment significantly enhanced CD31 (Figures 4A,B; *P* < 0.05). In addition, the expressions of the integral membrane of ZO-1, occludin, and claudin-5 proteins decreased in the peri-infarct regions. However, compared to the vehicle group, the vx-765-treated group increased the expression of these proteins (Figures 4C,D; *P* < 0.05). Immunoblotting images revealed comparable findings. On the other hand, immunoblots revealed that the expressions of GLUT-1 and OPN proteins, important in maintaining the structural integrity of BBB, decreased following MCAO. However, treatment with vx-765 reversed the MCAO-induced GLUT-1 and OPN degradation at the peri-infarct edge (Figures 4E,F; *P* < 0.05).

### vx-765 Increased the Encapsulation Coverage of Pericytes and NeuN-Positive Cells in Peri-infarct Regions

**Figure 5:**
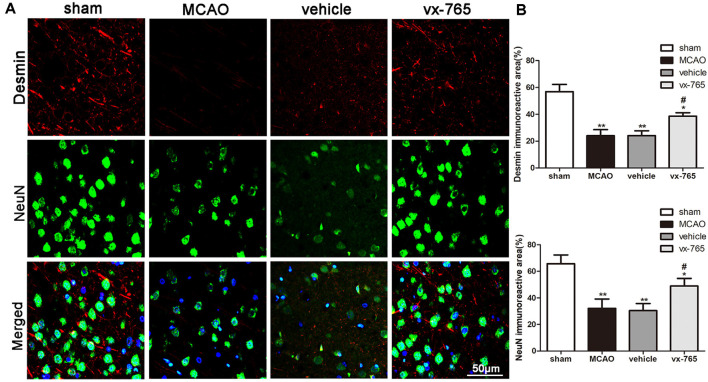
Encapsulation rate of pericytes and NeuN-positive cells in peri-infarct regions after caspase-1 inhibition by vx-765. **(A)** Representative immunofluorescence pictures of desmin (red) and NeuN-positive cells (green) in the peri-infarct zone. Scale bar = 50 μm. **(B)** Fluorescence intensity and the corresponding statistics. Data are expressed as means ± SEM, *P < 0.05 and **P < 0.01, vs. sham; ^#^*P* < 0.05, vs. vehicle, *n* = 6 per group.

Encapsulation coverage of pericytes was evaluated by the desmin immunofluorescence in peri-infarct regions of rats. Overall, the immunoreactivity of desmin was significantly decreased in MCAO animals compared to that in controls. However, compared to the vehicle group, the vx-765-treated group significantly enhanced the encapsulation coverage of pericytes (Figures 5A,B; *P* < 0.05). Similarly, the number of NeuN-positive cells in MCAO animals was significantly low compared to that in the sham-operated animals. However, vx-765 treatment significantly increased NeuN-positive cells in the experimental group compared vehicle animals (Figures 5A,B; *P* < 0.05), suggesting that vx-765 could alleviate MCAO-induced neuron damage by increasing the encapsulation rate of pericytes and NeuN-positive cells.

### vx-765 Reduced MMPs and TIMPs After Cerebral Infarction

**Figure 6:**
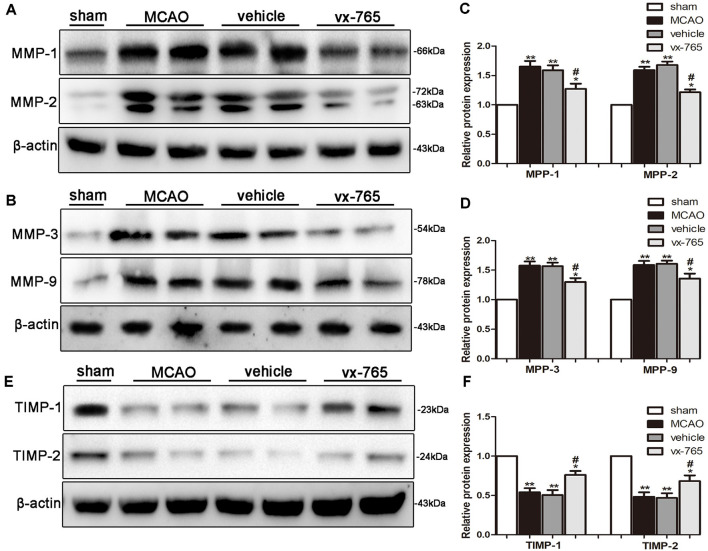
Expression of MMPs and upregulation of TIMPs in the peri-infarct area after caspase-1 inhibition by vx-765. **(A,B)** Western blot of matrix metalloproteinase (MMP)-1, MMP-2, MMP-3, MMP-9, and β-actin proteins in brain tissues and** (C,D)** corresponding statistics. **(E)** Western blotting on the expression of tissue inhibitor of metalloproteinases (TIMP)-1, TIMP-2, and β-actin proteins with **(F)** corresponding statistics. Data are expressed as means ± SEM, *P < 0.05 and **P < 0.01, vs. sham; ^#^*P* < 0.05, vs. vehicle, *n* = 6 per group.

The level of MMPs significantly increased at the peri-infarct edge of experimental rats compared to controls. Immunoblotting analysis revealed that vx-765-treated rats displayed a significant decrease in the level of MMPs at the peri-infarct edge compared with the vehicle group (Figures 6A–D; *P* < 0.05). TIMPs such as TIMP-1 and TIMP-2 are members of MMP tissue endogenous inhibitors. They inhibit functioning of MMPs by directly binding to the catalytic zinc cofactors. vx-765 treatment inhibited infarction-induced TIMP-1 and TIMP-2 degradation after MCAO, in contrast to the vehicle group (Figures 6E,F; *P* < 0.05). These findings suggest that vx-765 could ameliorate MCAO-induced BBB injury by suppressing the MMPs and upregulating the TIMPs.

### vx-765 Reduced Pyroptosis of Associated Molecules After MCAO

**Figure 7:**
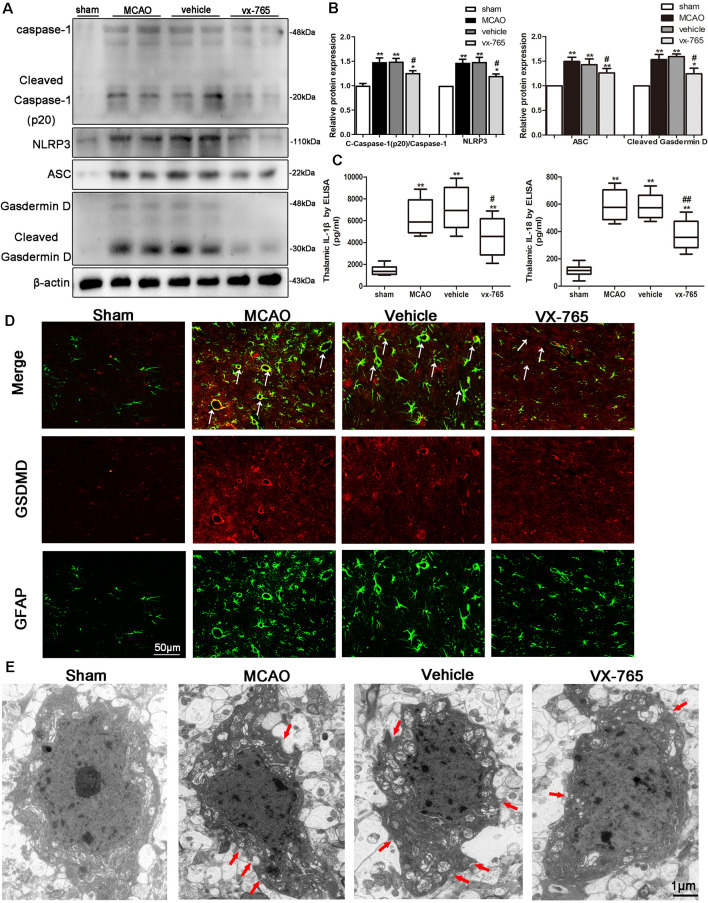
Expression of pyroptosis-related molecules in peri-infarct regions after caspase-1 inhibition by vx-765. **(A)** Western blot bands on caspase-1, nucleotide-binding domain (NOD)-like receptor family pyrin domain containing 3 (NLRP3), apoptosis-related speckle-like protein (ASC), Gasdermin D (GSDMD), and β-actin proteins and **(B)** corresponding statistical analyses, *n* = 6 rats per group. **(C)** The levels of interleukin-1β (IL-1β) and IL-18 in peri-infarct tissue were estimated using enzyme-linked immunosorbent assay (ELISA), *n* = 5 rats per group. **(D)** Double immunostaining of GFAP and GSDMD revealed a good co-localization of these two markers. Scale bar = 50 μm. The white arrow indicates the co-localization of GSDMD and GFAP. **(E)** Representative transmission electron microscopy images of astrocyte in the peri-infarct area. Red arrow head: membrane pores. Scale bar = 1 μm, *n* = 6 rats per group. All data are expressed as means ± SEM, *P < 0.05 and **P < 0.01, vs. sham; ^#^*P* < 0.05 and ^##^*P* < 0.01, vs. vehicle.

Caspase-1 is activated by inflammasomes, triggering pyroptosis, a programmed necrosis. Pyroptosis is a lytic type of programmed cell death that induces inflammatory response. Assembly and activation of NLRP3 inflammasome activate caspase-1 and induce secretion of IL-1β and IL-18 (Broz and Dixit, 2016). The effects of vx-765 inhibitor on pyroptosis-related molecules after MCAO were explored to investigate the protective effects of caspase-1 inhibition in cerebral infarction damage. Accordingly, ischemic stroke was found to significantly increase the levels of cleaved caspase-1, ASC, NLRP3, cleaved GSDMD, IL-1β, and IL-18. However, these levels were abrogated by vx-765 treatment with (Figures 7A–C; *P* < 0.05), collectively suggesting that activation of caspase-1 enhances the expression of proteins in components of NLRP3/ASC/GSDMD pathways. Consistently, double immunofluorescence showed that compared to controls, rats treated with vx-765 showed inhibited secretion of GSDMD in astrocytes in the peri-infarct region after MCAO, 3 days after stroke (Figure 7D), validated by findings of a transmission electron microscope (Figure 7E), in which membrane pores were less frequent in vx-765-treated, compared to vehicle-treated, MCAO rats. These findings further suggested that inhibiting caspase-1 could mitigate MCAO-induced BBB damage by inhibiting the inflammasome-mediated release of cytokines.

### vx-765 Protected Against BBB Dysfunction by Suppressing the RAGE/MAPK Pathway

**Figure 8:**
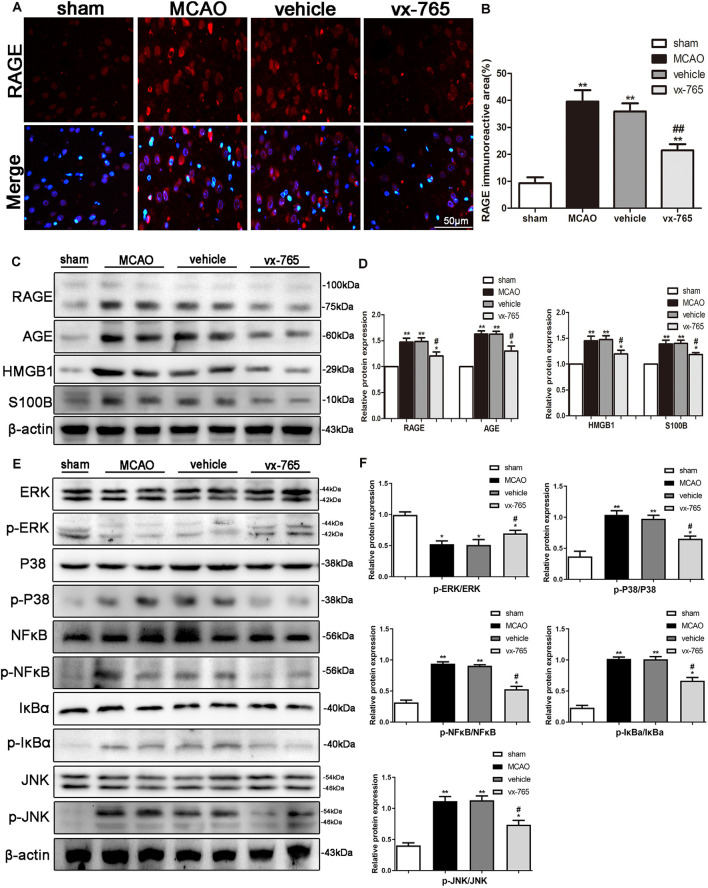
BBB function after inhibition of the receptor for advanced glycation end products (RAGE)/mitogen-activated protein kinase (MAPK) pathway after MCAO. **(A)** Representative immunofluorescence images of RAGE staining (red) in the peri-infarct regions. Scale bar = 50 μm. **(B)** Fluorescence intensity and the corresponding data analysis. **(C)** Western blot on the expression of RAGE, AGE, high-mobility family box-1 protein (HMGB1), S100B, and β-actin, with **(D)** the corresponding statistics. **(E)** Protein expressions of p-ERK/ERK, p-P38/P38, p-JNK/JNK, p-NF-κB/NF-κB, p-IκBa/IκBa, and β-actin as detected by western blotting, with **(F)** corresponding statistics. All data are expressed as means ± SEM, *P < 0.05 and **P < 0.01, vs. sham; ^#^*P* < 0.05 and ^##^*P* < 0.01, vs. vehicle, *n* = 6 per group.

RAGE is a member of the RAGE/MAPK family associated with neuron survival. Activation of RAGE exacerbates stroke injury whereas P38 signaling aggravates stroke-induced inflammation. We studied the expression of inflammation factors of RAGE, HMGB1, AGE, and S100B after cerebral infarction in rats treated with vx-765 to better understand the mechanism of RAGE in BBB damage. Quantitative immunoreactivity results revealed that expression of RAGE significantly increased in MCAO animals compared to controls. However, vx-765 treatment significantly decreased expression of RAGE compared to the vehicle animals (Figures 8A,B; *P* < 0.05). Further to this, the levels of RAGE, HMGB1, AGE, and S100B were significantly higher in the MCAO group than in the controls (Figures 8C,D). However, these levels were significantly lower in the vx-765-treated animals compared to the vehicle animals (*P* < 0.05).

Activated MAPKs can induce the release of NF-κB dimers from the inactive cytoplasmic NF-κB complex, thus prompting the nuclear translocation of NF-κB. As such, we investigated the effect of vx-765 treatment on the p-P38, p-JNK, p-ERK, and p-NF-κB pathways. We found that the expression levels of p-JNK, p-P38, p-NF-κB and p-IκBa were higher in the MCAO animals than in controls. However, the expression of these proteins significantly decreased in the vx-765-treated animals compared to the vehicle group. Moreover, the level of p-ERK expression was significantly higher in the vx-765-treated group than in the vehicle group (Figures 8E,F; *P* < 0.05). Together, these findings demonstrated that vx-765 treatment attenuated the inflammation-induced BBB damage by inhibiting the RAGE/MAPK pathway.

## Discussion

**Figure 9:**
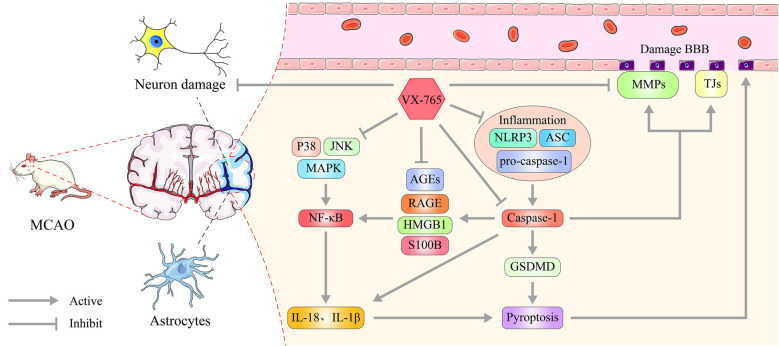
Effect of caspase-1 inhibition on ischemia and neuronal damage. Caspase-1 is generated and activated by NLRP3 inflammasome, ASC, and pro-caspase-1 following cerebral infarction. And it matures IL-1β and IL-18 and triggers GSDMD-induced pyroptosis, which facilitates increased MMPs and decreased TJ proteins, TIMP expression, and changes in BBB permeability. vx-765 improved the ischemia-induced BBB disruption through upregulating the expression of TJ proteins and TIMPs and suppressing the expression of MMPs, modulating the pyroptosis activation and RAGE/MAPK pathway.

In this study, we used rat models to explore the effect of vx-765, a caspase-1 inhibitor, on BBB dysfunction after MCAO-induced cerebral ischemia. We found that vx-765 treatment reduced cerebral ischemic injury and cerebral edema after MCAO. It further modulated neurological deficits, histopathological damage, and the encapsulation of pericytes. Moreover, vx-765 treatment reduced the leakage of the endothelial barrier and enhanced the expression of TJ proteins, AJ proteins, and TIMPs. It further modulated caspase-1 and the secretion of NLR inflammasome together with MMPs and RAGE/MAPK pathways at the peri-infarct regions after MCAO injury. The increasing global burden of ischemic stroke has led to an unmet need for effective prevention strategies (Macrez et al., 2011). This study strongly demonstrated that vx-765 is an effective and promising neuroprotectant against ischemic stroke (Figure 9).

BBB is composed of specialized ECs, linked by complex TJs and AJs. These cells are also surrounded by astrocytes and pericytes. Normal BBB is structurally specialized to prevent paracellular transport of multiple hydrophilic compounds across the cerebral endothelium, thus hindering extravasation migration of blood-borne cells into the central nervous system (CNS; Moretti et al., 2015). Meanwhile, inflammasome is an oligomer composed of three proteins: intracellular pattern recognition receptors (PRRs), linker apoptosis-related speckle-like protein (ASC), and pro-caspase-1. This oligomer activates caspase-1 downstream. At the same time, the activated caspase-1 can induce cell membrane perforation, thus causing leakage of cellular constituents. This leads to pyroptosis, a programmed cell death associated with inflammation (Latz, 2010). In the ischemic stroke model, inhibition of caspase-1 or caspase-1 knockout reduced cerebral infarction damage and significantly protected brain injury (Friedlander et al., 1997; Hara et al., 1997). vx-765 is a VRT-043198 prodrug that inhibits caspase-1 against permeation of BBB (Boxer et al., 2010). On oral administration, vx-765 was shown to be efficiently converted to VRT-043198, effectively inhibiting lipopolysaccharide-induced cytokine secretion in rats (Wannamaker et al., 2007). vx-765 alleviates inflammatory response in neurodegenerative diseases and improves Aβ deposition and cognitive deficits in Alzheimer’s disease models (Flores et al., 2018). In this study, we found that expression of caspase-1 in neurons was increased at the peri-infarct regions after MCAO. Caspase-1 activation in neurons was induced by MCAO, leading to neuronal damage and neuroinflammation, thereby activating the release of inflammatory factors by astrocytes. This promotes the expression of inflammatory factors and the membrane-breaking protein GSDMD. This ruptures the cell membrane, inducing undesirable BBB permeability. However, vx-765 restored effective BBB semipermeability after cerebral infarction, perhaps by inhibiting the release of inflammatory factors in pyrolysis of astrocytes, as well as inhibiting the invasion of peripheral immune cells into the CNS. The secreted inflammatory mediators, including IL-1β and IL-18, recruit immune cells and stimulate IL-mediated expression and activation of adhesion molecules on ECs. These pro-inflammatory molecules also trigger dynamic recombination of the junction complex between ECs, thereby changing the permeability of BBB. These findings suggest that vx-765 is a novel cytokine inhibitor, potentially treating damaged BBB after ischemia. Herein, caspase-1 inhibitor vx-765 protected peri-infarct region against hypoxia damage and ameliorated neurological dysfunction after ischemia.

Infarct injury severely damages the BBB, increasing its permeability. This allows undesirable extravasation of multiple blood components, thereby increasing brain tissue damage (Prakash and Carmichael, 2015; Hu et al., 2017). In addition, infarct damage aggravates the permeability of the BBB by inhibiting the expression levels of TJ, AJ, GLUT-1, and OPN proteins. Meanwhile, BBB dysfunction is central to ischemic cerebrovascular disease (Won et al., 2014; De Fusco et al., 2017; Tang et al., 2017). With this in mind, maintaining the integrity of the BBB is considered a new treatment strategy for ischemic stroke (Jiang et al., 2018). ZO-1, occludin, and claudin-5 are the major structural barrier proteins of the BBB and are thus considered as sensitive indicators of normal and dysfunctional BBB (Sladojevic et al., 2019). Denaturation of these proteins increases the paracellular permeability of BBB (Sandoval and Witt, 2008). In this study, vx-765 treatment significantly attenuated EB leakage and suppressed brain edema in MCAO-induced rats. There was a significant upregulation of TJ proteins including claudin-5, ZO-1, and occludin; AJ proteins; GLUT-1 proteins; and OPN proteins following vx-765 treatment in MCAO-induced rats compared to the vehicle groups. These results strongly suggested that vx-765 could effectively enhance the integrity and function of BBB.

Tang et al. (2017) reported that under cerebral infarction, MMPs such as MMP-1, MMP-2, MMP-3, and MMP-9 were overexpressed in rodents and nonhuman primates during early stages of brain injury. MMP-2/MMP-9 aggravates BBB dysfunction caused by cerebral infarction by degrading ZO-1 and claudin-5, all TJ proteins (Liu et al., 2012). Treatment with MMP inhibitors or MMP-neutralizing antibodies reduced the severity of infarction and prevented BBB destruction after ischemic stroke. MMP inhibitors can also significantly block the degradation of claudin-5 and angiotensin after focal cerebral infarction, thus alleviating BBB damage in the early periods of ischemic stroke (Benjamin and Khalil, 2012). Activated MMPs cause the TIMPs and are bound by TIMPs, thus limiting the effectiveness of the MMP-2/MMP-9 matrix (Yang and Rosenberg, 2015). In this study, vx-765 treatment enhanced the expression levels of TIMP-1 and TIMP-2 but reduced the expression levels of MMP-1, MMP-2, MMP-3, and MMP-9 proteins after cerebral infarction. Meanwhile, vx-765 therapy obliterated the initial effects on MMP-1 and MMP-2 after MCAO. After MMP activation, TIMPs bind to the corresponding active site and block substrate availability, thus inhibiting MMP-9 (TIMP-2 appears to inhibit MMP-2). vx-765 increased the expression of TIMP-1 but inhibited MMP-9 functions after MCAO.

Inflammation is an important mechanism in the pathology process of cerebral infarction (Dziedzic, 2015; Kawabori and Yenari, 2015). BBB dysfunction increases the risk of bleeding and impairs restoration of brain tissues and neurological functions (Pan and Kastin, 2007). Furthermore, brain damage can lead to inflammasome-mediated pyroptosis of microvascular ECs (Rochfort and Cummins, 2015). However, vx-765, a caspase-1 inhibitor, can alleviate brain damage by reducing the expression of the inflammatory cleaved caspase-1 and key downstream pro-inflammatory cytokines (such as IL-1β and IL-18). It further prevents secretion of GSDMD and aggregation of ASC of microvascular ECs (Ge et al., 2018). In this study, vx-765 treatment reduced the expression of pyroptosis-related molecules such as NLRP3, ASC, GSDMD, IL-1β, and IL-18. It also suppressed the inflammatory response of BBB damage caused by cerebral infarction.

RAGE damages BBB during multiple human brain diseases such as cerebral infarction, Alzheimer’s disease, and multiple sclerosis. RAGE knockdown in rats reduces undesirable inflammatory reactions and BBB permeability after brain injury (Wan et al., 2015). Moreover, several studies have reported that MAPK, ERK1/ERK2, JNK, and NF-κB play important roles in ischemic stroke injury. More specifically, JNK expression aggravates ischemic stroke injury by inducing neuronal apoptosis (Cui et al., 2007; Joo et al., 2007; Nithianandarajah-Jones et al., 2012). In this study, vx-765 treatment inhibited the expression of RAGE- and MAPK-related pathway molecules. The interactions between RAGE and MAPK during ischemic stroke promoted the expression of MMP-2 and MMP-9. On the other hand, increased permeability of BBB mediated by MMP-2 and MMP-9, the reduction in P38 and JNK levels, and the increase in ERK-mediated signaling in ECs were reversed by vx-765. These findings imply that vx-765 prevents deleterious effects of caspase-1 by inhibiting RAGE signaling and the subsequent activation of P38, JNK, and NF-κB as well as promoting ERK phosphorylation. We postulate that after MCAO, vx-765 reduces the expression of inflammatory factors and reverses the permeability of BBB by inhibiting the MAPK and RAGE pathways. Elsewhere, vx-765 was found to ameliorate brain damage and undesirable microglial inflammatory response by inhibiting the NF-κB signaling pathway (Li et al., 2019). Our findings notwithstanding, this research suffered from several limitations; first, we did not assess the expression of other pyroptosis-associated molecules such as NLRP3, ASC, and GSDMD after MCAO. Second, we did not microscopically observe the morphological changes in BBB after vx-765 treatment. Taken altogether, our findings demonstrated that vx-765 can positively modulate the inflammation response after BBB damage, probably mediated by inhibition of RAGE and MAPK pathways. Currently, there is limited knowledge regarding the role of vx-765 in modulating undesirable inflammatory response. As such, these results broaden and enrich our knowledge on the potential benefits of vx-765 in preventing and treating MCAO-induced BBB damage.

## Conclusion

Taken together, inhibition of caspase-1 significantly ameliorates ischemia-associated BBB injury and integrity by suppressing the activation of pyroptosis as well as the RAGE/MAPK pathway.

## Data Availability Statement

All datasets generated for this study are included in the article.

## Ethics Statement

The animal study was reviewed and approved by the Animal Ethics Committee of Jinan University.

## Author Contributions

YL, WC, and PS performed the experiments and illustrated the experimental results. YL, MH, and YZ conceived the idea and designed the animal experiments. RL, XX, YZ, and PZ participated in the study design and data analysis. JS, RL, and JX participated in tissue section analysis and neurological behavioral analysis. YL, MH, and YZ wrote the manuscript. All authors read and approved the current version of the manuscript.

## Conflict of Interest

The authors declare that the research was conducted in the absence of any commercial or financial relationships that could be construed as a potential conflict of interest.

## Abbreviations

MCAO: middle cerebral artery occlusion
BBB: blood–brain barrier
CBF: cerebral blood flow
EB: evans Blue
ZO-1: zonula occludens-1
TJs: tight junctions
AJs: adherens junctions
MMPs: matrix metalloproteinase proteins
TIMPs: tissue inhibitor of metalloproteinases
NLRP3: nucleotide-binding domain (NOD)-like receptor family pyrin domain containing 3
ASC: apoptosis-related speckle-like protein
GSDMD: Gasdermin D
IL-1β: interleukin-1β
IL-18: interleukin-18
RAGE: receptor for advanced glycation end
AGE: advanced glycation end products
HMGB1: high-mobility family box-1 protein
MAPK: mitogen-activated protein kinase
JNK: c-Jun N-terminal kinase
ERK: extracellular regulated protein kinases
NF-κB: nuclear factor-κB.
